# A literature overview of age- and Tanner stage-specific reference intervals for sex hormones for children aged 0–18 years

**DOI:** 10.1530/JME-26-0037

**Published:** 2026-08-21

**Authors:** Anouk Olthof, Anita Boelen, Jacquelien J Hillebrand, Sabine E Hannema, Annemieke C Heijboer

**Affiliations:** ^1^ Endocrine Laboratory, Department of Laboratory Medicine, Amsterdam UMC Location University of Amsterdam, Amsterdam, the Netherlands; ^2^ Endocrine Laboratory, Department of Laboratory Medicine, Amsterdam UMC Location Vrije Universiteit Amsterdam, Amsterdam, the Netherlands; ^3^ Amsterdam Gastroenterology, Endocrinology & Metabolism, Amsterdam, the Netherlands; ^4^ Amsterdam Reproduction and Development, Amsterdam, the Netherlands; ^5^ Department of Pediatric Endocrinology, Emma Children’s Hospital, Amsterdam UMC location University of Amsterdam, Amsterdam, the Netherlands; ^6^ Department of Pediatric Endocrinology, Amsterdam UMC location Vrije Universiteit Amsterdam, Amsterdam, the Netherlands

**Keywords:** adolescents, gonadal steroid hormones, gonadotropins, reference values, puberty

## Abstract

This study aims to provide a comprehensive overview of age- and Tanner stage-specific reference intervals (RIs) for testosterone, estradiol, luteinizing hormone (LH), follicle-stimulating hormone (FSH), and sex hormone-binding globulin (SHBG) in children and adolescents and to highlight the importance of age- and Tanner stage-specific RIs for clinical practice. A literature review was performed to identify studies reporting RIs on testosterone and estradiol quantified using GC–MS/MS or LC–MS/MS and LH, FSH, and SHBG quantified using immunoassays in healthy individuals aged 0–18 years, categorized by age and/or Tanner stage. Sample sizes were evaluated according to the Clinical Laboratory Standards Institute (CLSI) guideline CLSI EP28-A3c, with ≥120 samples per group being acceptable, 39–119 samples being poor, and ≤38 samples being unacceptable. Analytical methods and statistical approaches were carefully documented. Fourteen studies were included for testosterone, seven for estradiol, thirteen for LH and FSH, and nine for SHBG. The results revealed considerable variability in the availability and quality of RIs, with many studies lacking adequate sample size or appropriate partitioning. Testosterone, estradiol, LH, and FSH RIs demonstrated significant changes during mini-puberty and puberty, with clear sex-specific patterns. SHBG RIs fluctuated throughout childhood and adolescence. This literature review demonstrates that RIs for HPG axis hormones show significant changes during mini-puberty and puberty, although data using appropriate analytical methods, using sufficient sample sizes, and reporting on age categories, Tanner stage, and menstrual cycle phase are scarce. Robust age- and Tanner stage-specific RIs are essential to improve diagnostic accuracy and individualized care in children and adolescents.

## Introduction

Sex hormone production is initiated in early fetal life and continues throughout life, with sex- and age-specific changes that characterize crucial developmental events, such as mini-puberty (1, 2), adrenarche, puberty (3), pregnancy, and menopause (4, 5). Estradiol and testosterone are key biomarkers of sexual development, supporting the maintenance of female and male phenotypes (6). Luteinizing hormone (LH) and follicle-stimulating hormone (FSH) are essential for gonadal regulation and are critical markers for diagnosing pituitary and reproductive dysfunctions, as well as early or delayed puberty (7). Sex hormone-binding globulin (SHBG) modulates the bioavailability of estradiol and testosterone throughout childhood and adolescence (8).

In case of suspected pathologies, such as ambiguous genitalia or other differences of sex development, delayed, precocious, or abnormal course of puberty, hypogonadism, oligomenorrhea, amenorrhea, hirsutism, and polyendocrine metabolic ovarian syndrome (PMOS), measurement of these hormones is crucial (9, 10, 11, 12). To accurately interpret the results of these hormone measurements, reference intervals (RIs) are essential. Robust, age- and Tanner stage-specific RIs, partitioned by sex, are essential to differentiate between normal and pathological states and to guide appropriate clinical follow-up.

In current clinical practice, many laboratories have not established their own RIs, especially not for the pediatric population. In addition, RIs are often not appropriately defined, that is, many lack suitable age- or Tanner stage-specific groups. As a result, pediatric RIs are often not incorporated in Laboratory Information Systems and Electronic Health Records. This presents a significant opportunity for improvement, as appropriate RIs are important for the timely diagnosis and management of pathologies, particularly in children. Therefore, we recognize the need to summarize the literature on pediatric RIs for hormones related to the hypothalamic–pituitary–gonadal axis (HPG axis).

Because the sex hormones testosterone and estradiol are present in circulation in low concentrations, analytical methods used for their quantification require high sensitivity and specificity. Mass spectrometry-based methods, known for their sensitivity and specificity, are preferred for measuring these hormones, as opposed to immunoassays which are well suited for LH, FSH, and SHBG. Consequently, RIs established using mass spectrometry-based methods are of primary interest for these hormones (13).

Therefore, the aim of this review is to summarize the current literature on age- and Tanner stage-specific RIs for testosterone and estradiol quantified using mass spectrometry-based methods and age- and Tanner stage-specific RIs for LH, FSH, and SHBG quantified using immunoassays in children aged 0–18 years.

## Methods

### Inclusion criteria for studies

RIs on testosterone, estradiol, LH, FSH, and SHBG were collected from the literature, based on age categories (birth to 18 years) and Tanner stages 1–5 in children aged approximately 5 years and older. Literature was collected through a PubMed search (https://pubmed.ncbi.nlm.nih.gov/). Studies were included if they reported RIs categorized for either age (0–18 years), Tanner stage, or both, for at least one of these five hormones in a healthy population (e.g. individuals without known endocrine disorders or use of hormonal therapy). Studies were included in this review regardless of the method used to determine the Tanner stages, i.e. pubic hair growth (PH), testicular volume (TV), genital stage (G), or breast stage (B). TV was correlated to a corresponding Tanner G definition. For testosterone and estradiol, only studies that quantified the concentrations using mass spectrometry-based methods such as liquid chromatography–tandem mass spectrometry (LC–MS/MS) or gas chromatography–MS/MS (GC–MS/MS) were included. For LH, FSH, and SHBG, studies using any sort of immunoassay were included.

### Data collection and analysis

Sample sizes for the RIs in each study were evaluated according to the CLSI guideline EP28-A3c, with groups of ≥120 samples considered acceptable, 39–119 samples poor, and ≤38 samples unacceptable (14). Key factors that were documented included specific assay type (name and company) used for hormone quantification, the range of the intervals reported, and the statistical methods used to calculate the RIs.

### Statistical calculations

For steroids and SHBG, concentrations were converted to recommended SI units (nmol/L or pmol/L) when presented in a conventional unit. Data were collected in Microsoft Excel to create a broad overview. Bar graphs to visualize RIs were created using GraphPad Prism 10.6.0 for Windows.

## Results

**Figure 1:**
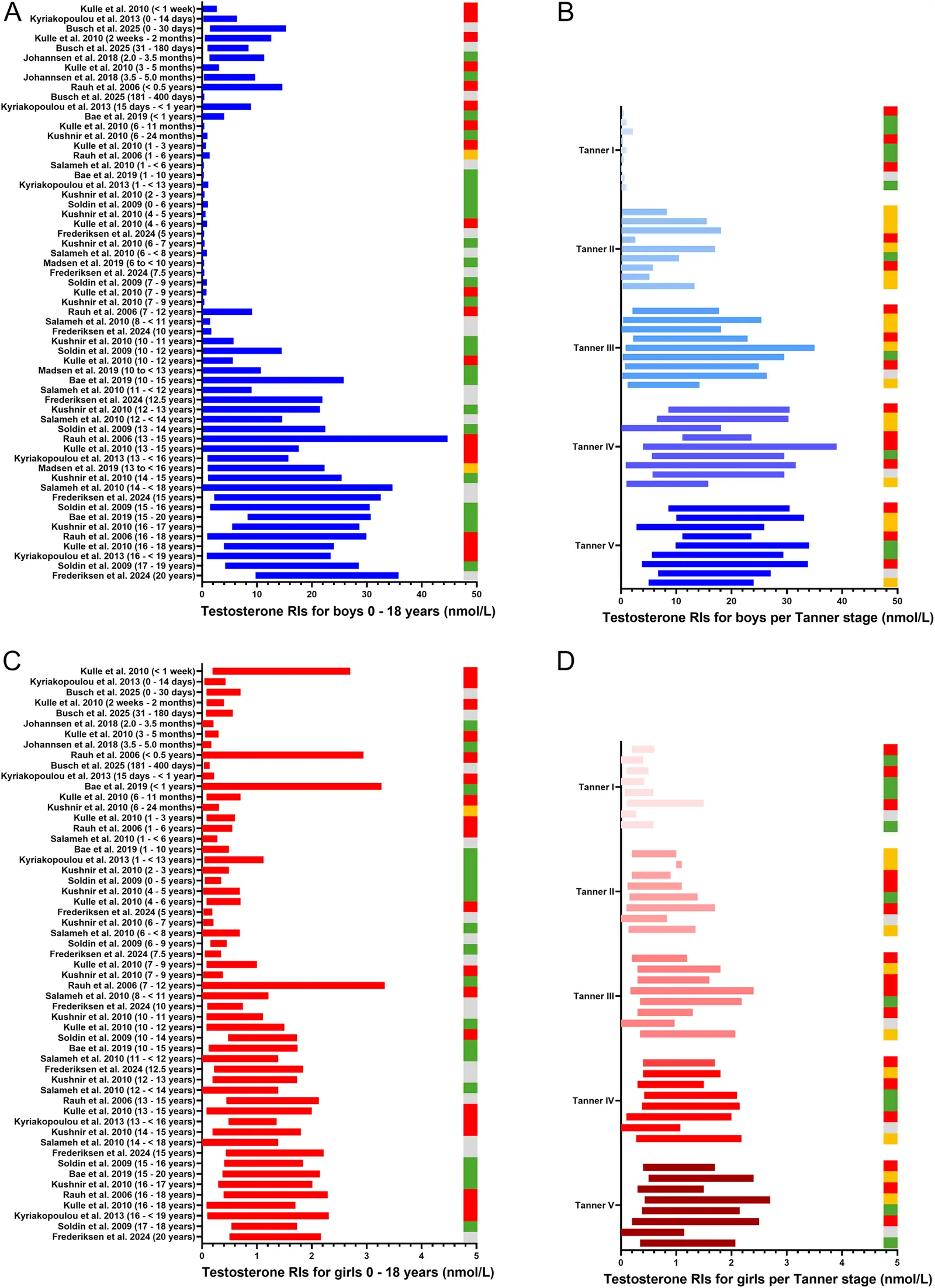
Age- and Tanner stage-specific RIs for testosterone in children using LC–MS/MS. (A) Age-specific RIs for testosterone in boys. (B) Tanner stage-specific RIs for testosterone in boys. Top-down per Tanner stage: Ankarberg-Lindgren *et al.* (30), Bae *et al.* (21), Madsen *et al.* (28), Ankarberg-Lindgren *et al.* (27) (GC–MS/MS), Søeborg *et al.* (10), Kushnir *et al.* (26), Kulle *et al.* (25), Salameh *et al*. (15), and Kushnir *et al.* (42). (C) Age-specific RIs for testosterone in girls. (D) Tanner stage-specific RIs for testosterone in girls. Top-down per Tanner stage: Ankarberg-Lindgren *et al.* (30), Bae *et al.* (21), Ankarberg-Lindgren *et al.* (27) (GC–MS/MS), Søeborg *et al.* (10), Kushnir *et al.* (26), Kulle *et al.* (25), Salameh *et al.* (15), and Kushnir *et al.* (42). Sample size indication: red: ≤38; orange: 39–119; green: ≥120; gray: no information available.

**Figure 2:**
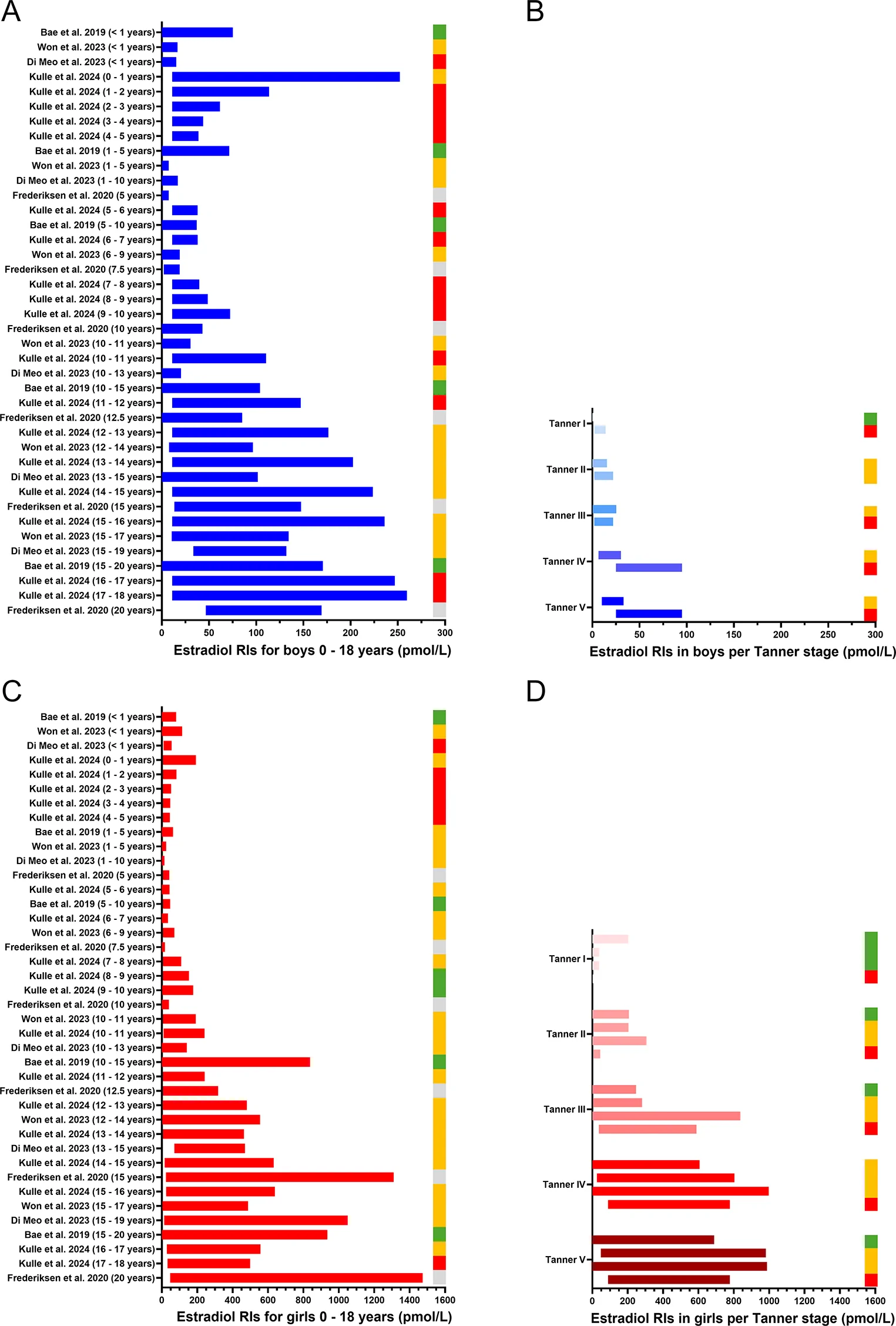
Age- and Tanner stage-specific RIs for estradiol in children using LC–MS/MS. (A) Age-specific RIs for estradiol in boys. (B) Tanner stage-specific RIs for estradiol in boys. Top-down per Tanner stage: Bae *et al.* (21) and Ankarberg-Lindgren *et al.* (27) (GC–MS/MS). (C) Age-specific RIs for estradiol in girls. (D) Tanner stage-specific RIs for estradiol in girls. Top-down per Tanner stage: Kulle *et al.* (9), Madsen *et al.* (31), Bae *et al.* (21), and Ankarberg-Lindgren *et al.* (27) (GC–MS/MS). Sample size indication: red: ≤38; orange: 39–119; green: ≥120; gray: no information available.

**Figure 3:**
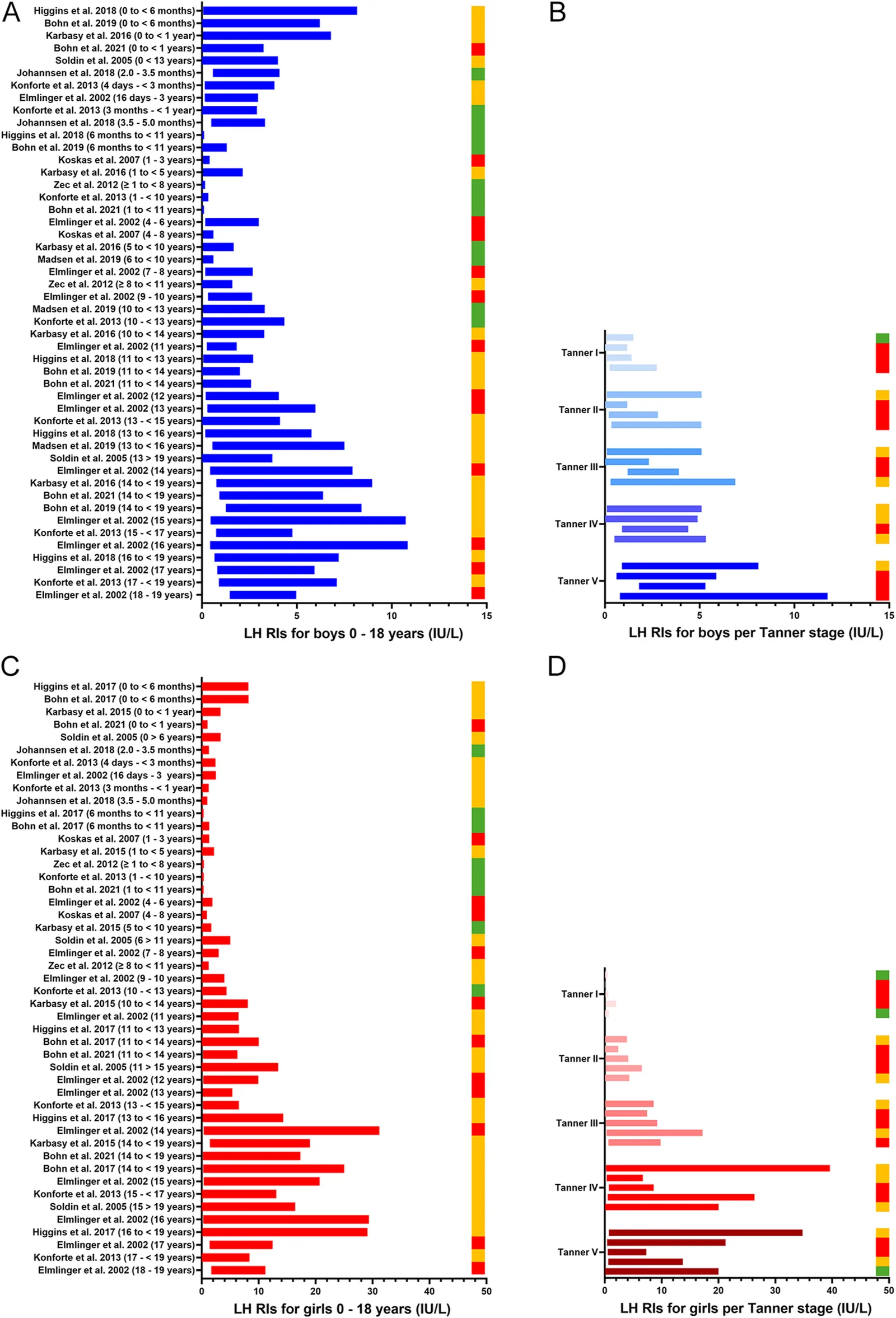
Age- and Tanner stage-specific RIs for LH in children using LC–MS/MS. (A) Age-specific RIs for LH in boys. (B) Tanner stage-specific RIs for LH in boys. Top-down per Tanner stage: Madsen *et al.* (28), Konforte *et al.* (19), Koskas *et al.* (36), and Elmlinger *et al.* (20). (C) Age-specific RIs for LH in girls. (D) Tanner stage-specific RIs for LH in girls. Top-down per Tanner stage: Madsen *et al.* (28), Konforte *et al.* (19), Koskas *et al.* (36), Elmlinger *et al.* (20), and Sehested *et al.* (34). Sample size indication: red: ≤38; orange: 39–119; green: ≥120; gray: no information available.

**Figure 4:**
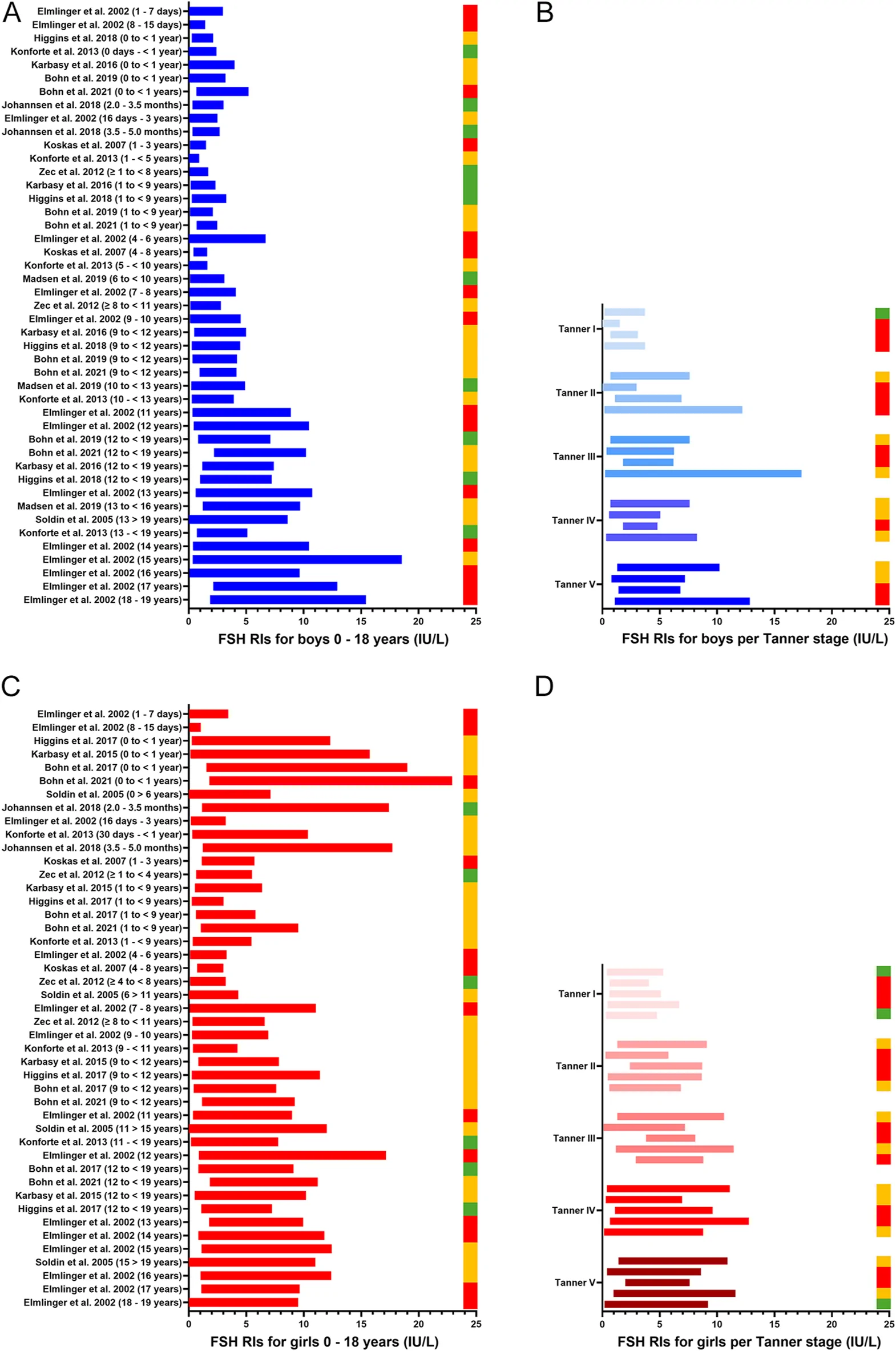
Age- and Tanner stage-specific RIs for FSH in children using LC–MS/MS. (A) Age-specific RIs for FSH in boys. (B) Tanner stage-specific RIs in boys. Top-down per Tanner stage: Madsen *et al.* (28), Konforte *et al.* (19), Koskas *et al.* (36), and Elmlinger *et al.* (20). (C) Age-specific RIs for FSH in girls. (D) Tanner stage-specific RIs in girls. Top-down per Tanner stage: Madsen *et al.* (31), Sehested *et al.* (34), Konforte *et al.* (19), Koskas *et al.* (36), and Elmlinger *et al.* (20). Sample size indication: red: ≤38; orange: 39–119; green: ≥120; gray: no information available.

**Figure 5:**
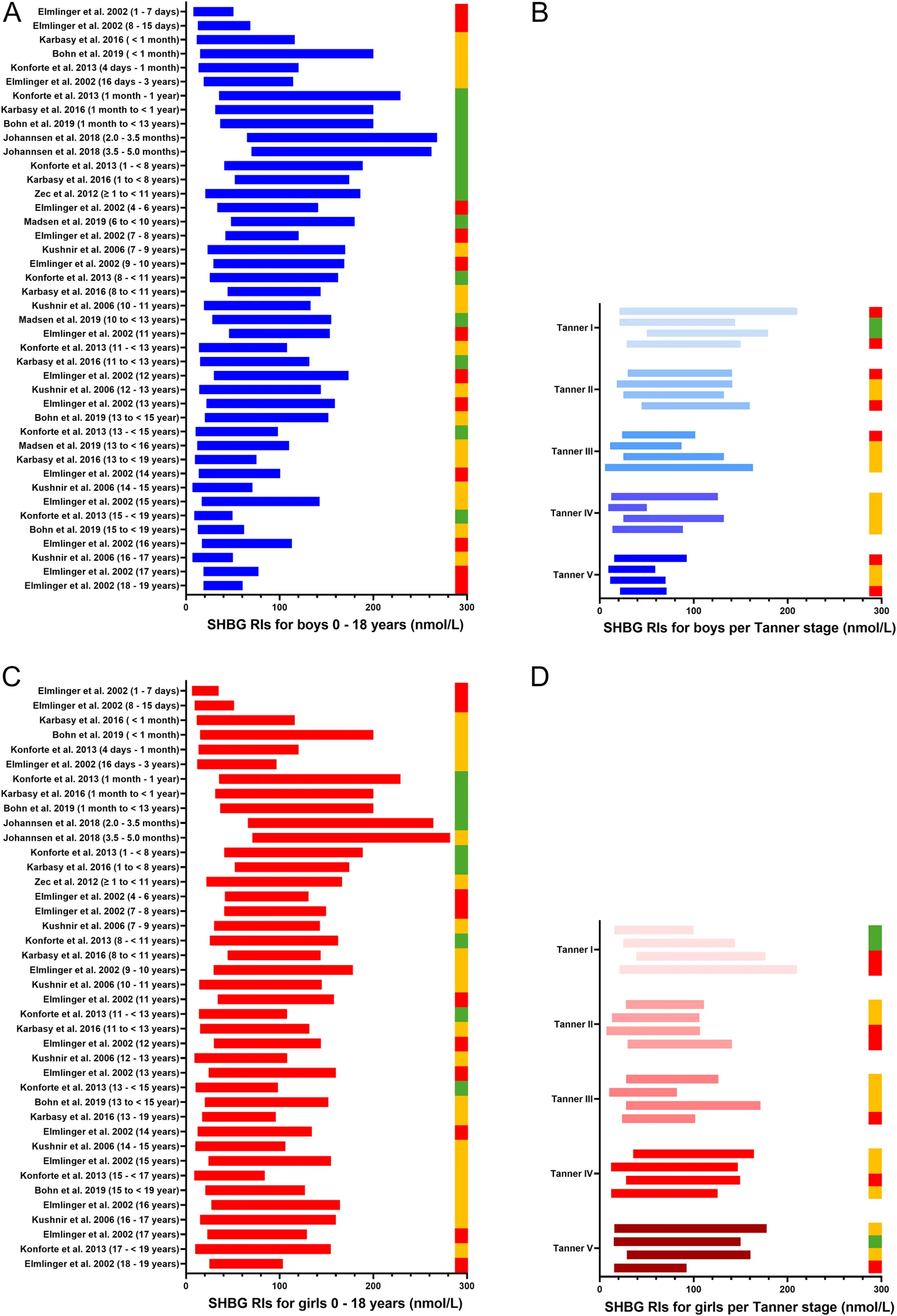
Age- and Tanner stage-specific RIs for SHBG in children. (A) Age-specific RIs for SHBG in boys. (B) Tanner stage-specific RIs in boys. Top-down per Tanner stage: Madsen *et al.* (31), Konforte *et al.* (19), Kushnir *et al.* (42), and Elmlinger *et al.* (20). (C) Age-specific RIs for SHBG in girls. (D) Tanner stage-specific RIs in girls. Top-down per Tanner stage: Madsen *et al.* (31), Konforte *et al.* (19), Kushnir *et al.* (42), and Elmlinger *et al.* (20). Sample size indication: red: ≤38; orange: 39–119; green: ≥120; gray: no information available.

All reference intervals per age or Tanner category were reported (Figs 1, 2, 3, 4, 5), regardless of sample size. Not all studies reported sample sizes (11, 15, 16, 17) or the specific age categories (11, 16). Some studies presented age categories exceeding 18 years of age (11, 16, 18, 19, 20, 21, 22, 23) (detailed information is provided in the Supplementary Tables, see section on Supplementary materials given at the end of this article).

For testosterone, the upper limit of the RI (ULN) in boys during mini-puberty (age 1–6 months) was approximately 10 nmol/L and decreased to undetectable in the years after (Fig. 1A). From around the age of 8 years and with increasing Tanner stage, testosterone RI gradually increased toward the male adult RI (Fig. 1A and B). In girls, only a slight increase of the ULN was observed for testosterone throughout the first 18 years of life and per Tanner stage (Fig. 1C and D). For estradiol, the ULNs for both boys and girls were slightly higher during mini-puberty than the years after, even though there were only a few studies reporting on this period (Fig. 2A and C). From the age of 8–10 and with increasing Tanner stage, estradiol RIs started to rise for both boys and girls, albeit covering different absolute concentrations (Fig. 2). During mini-puberty, the ULNs of LH were relatively high, compared to the RIs at 1–10 years for both boys and girls (Fig. 3A and C). It was observed that, at later Tanner stages, girls have higher LH concentrations than boys (Fig. 3D). In the first year of age, the ULNs of FSH were relatively high in girls, compared to ULNs at 1–10 years of age (Fig. 4C). After the first year, the ULN increased with age and increasing Tanner stage in boys, resulting in broader RIs (Fig. 4A and B). For SHBG, a rise in both the lower limit of the RIs and the ULNs from the first years of life was observed for both boys and girls (Fig. 5A and C). During pubertal development, categorized per Tanner stage, the RIs became smaller with a decrease in ULNs (Fig. 5B and D), particularly in boys (Fig. 5B).

Table 1 shows the mass spectrometry methods used to quantify testosterone and estradiol concentrations. The majority used LC–MS/MS methods, whereas two studies used gas chromatography (GC–MS/MS) for quantification. In Table 2, the specific immunoassays used to quantify LH, FSH, and SHBG concentrations are presented. The statistical methods to determine the RIs are presented in both tables as well. Most studies employed the nonparametric method when 120 samples or more were available and used the Robust method for sample sizes between 39 and 119, in accordance with the CLSI guideline EP28-A3c (14).

**Table 1:** Analytical methods for testosterone and estradiol quantitation and statistical methods for reference interval calculation.

Reference	Analytical method	Statistical method to determine RI
**Testosterone**		
Rauh *et al.* (24)	LC–MS/MS	Median and range (97.5th percentile)
Soldin *et al.* (18)	LC–MS/MS	Hoffmann approach
Salameh *et al.* (15)	HTLC–MS/MS	Segmented regression with a sigmoidal curve + SD function
Kulle *et al.* (25)	LC–MS/MS	Median and range
Kushnir *et al.* (26)	LC–MS/MS	Robust method
Kyriakopoulou *et al.* (23)	LC–MS/MS	≥120 samples nonparametric method ≥40 and <120 samples robust method
Søeborg *et al.* (10)	TurboFlow–LC–MS/MS	Generalized additive models for location, scale, and shape – ‘GAMLSS’ package for R
Ankarberg-Lindgren *et al.* (27)	GC–MS/MS	Median values and 5th to 95th percentiles
Madsen *et al.* (28)	LC–MS/MS	≥120 samples nonparametric method ≥40 and <120 samples robust method
Johannsen *et al.* (29)	LC–MS/MS	Hormone concentrations were logarithm (log)-transformed and reported as back-transformed geometric means and p2.5–97.5
Bae *et al.* (21)	LC–MS/MS	≥120 samples nonparametric method ≥40 and <120 samples robust method
Ankarberg-Lindgren *et al.* (30)	LC–MS/MS	Median values and ranges
Frederiksen *et al.* (16)	LC–MS/MS	Generalized additive models for location, scale, and shape – ‘GAMLSS’ package for R
Busch *et al.* (17)	LC–MS/MS	Generalized additive models for location, scale, and shape – ‘GAMLSS’ package for R
**Estradiol**		
Ankarberg-Lindgren *et al.* (27)	GC–MS/MS	Median values and 5th to 95th percentiles
Bae *et al.* (21)	LC–MS/MS	≥120 samples nonparametric method <120 samples robust method with Box–Cox transformation
Madsen *et al.* (31)	LC–MS/MS	≥120 samples nonparametric method ≥40 and <120 samples robust method *Continuous reference intervals: ‘ReferenceIntervals’ package in R, Bootstrap resampling*
Won *et al.* (32)	Ultra-high performance LC–MS/MS	≥120 samples nonparametric method ≥40 and <120 samples robust method
Di Meo *et al.* (22)	LC–MS/MS	≥120 samples nonparametric method ≥40 and <120 samples robust method
Kulle *et al.* (9)	UPLC–MS/MS	Median (M), the coefficient of variation (CV) (S), and the skewness (L) are utilized to model the changes according to age ‘LMS’ method by Cole and Green (33)
Frederiksen *et al.* (11)	LC–MS/MS	Generalized additive models for location, scale, and shape – ‘GAMLSS’ package for R

RI, reference interval.

**Table 2:** Analytical methods for LH, FSH and SHBG quantitation and statistical methods for reference interval calculation.

Article	Analytical method	Statistical method to determine RI
**LH, FSH and SHBG**		
Sehested *et al.* (34)	DELFIA (Wallac Oy, PerkinElmer)	Weighted regression quantiles (median and ±2 SD)
Elmlinger *et al.* (20)*	DPC Immulite and Immulite 2000® (DPC, Siemens Healthineers)	The central 95% range (between the 2.5 and the 97.5th percentiles)
Soldin *et al.* (35)*	IMMULITE 1000. (DPC, Siemens Healthineers)	Hoffmann approach
Koskas *et al.* (36)*	Enzyme-Linked Fluorescent Assay (ELFA) (BioMérieux)	Median and 10–90th percentiles (tables)
Zec *et al.* (37)	Cobas e411 (Roche Diagnostics)	Gaussian distribution: parametric method Non-Gaussian distribution: nonparametric Log-transformation +2.5 and 97.5th percentiles
Konforte *et al.* (19)	Architect i2000. (Abbott Diagnostics)	≥120 samples nonparametric method ≥40 and <120 samples robust method
Karbasy *et al.* (38)	UniCel DxC 800 Synchron (Beckman Coulter Diagnostics)	≥120 samples nonparametric method ≥40 and <120 samples robust method
Higgins *et al.* (39)*	VITROS 5600 IS (Ortho Clinical Diagnostics)	≥120 samples nonparametric method ≥40 and <120 samples robust method
Johannsen *et al.* (29)	AutoDELFIA (PerkinElmer)	Hormone concentrations were logarithm (log) transformed and reported as back-transformed geometric means and p2.5–97.5
Bohn *et al.* (40)	Cobas 8000 e602 (Roche Diagnostics)	≥120 samples nonparametric method ≥40 and <120 samples robust method
Madsen *et al.* (28)	IMMULITE 2000 XPi (Siemens Healthineers)	≥120 samples nonparametric method ≥40 and <120 samples robust method
Madsen *et al.* (31)	IMMULITE 2000 XPi (Siemens Healthineers)	≥120 samples nonparametric method ≥40 and <120 samples robust method *Continuous reference intervals: ‘ReferenceIntervals’ package in R, Bootstrap resampling*
Bohn *et al.* (41)*	Atellica® IM (Siemens Healthineers)	≥120 samples nonparametric method ≥40 and <120 samples robust method
Kushnir *et al.* (42)^†^	IMMULITE 2000 analyzer (DPC, Siemens Healthineers)	Nonparametric method

* Only LH + FSH.

^†^ Only SHBG.

## Discussion

This review summarized the current literature on RIs for testosterone and estradiol quantified using LC–MS/MS, and LH, FSH, and SHBG quantified using immunoassays in neonates, children, and adolescents. Eleven studies could be included for testosterone, six for estradiol, twelve studies for LH and FSH, and eight for SHBG.

Several studies included in our review are based on the Caliper study (43), a Canadian RI study that included age- and sex-specific RIs for various LH, FSH, or SHBG immunoassays, which adds further value to our analysis (19, 23, 38, 39, 40). Other studies were excluded from our overview because they reported reference curves across age as a continuous variable rather than providing age- or Tanner stage-specific RIs, although such continuous reference ranges are useful in clinical practice (44, 45, 46, 47, 48, 49, 50). However, participants in these studies were a selection from the cohorts of the Copenhagen Mini-Puberty Study (51, 52) or the Copenhagen Puberty Study (53, 54); both original Copenhagen studies were included in our overview.

In our overview, the RIs for both boys and girls clearly show a higher ULN for the hormones of the HPG axis during mini-puberty. The hormonal changes during mini-puberty have been well characterized in the literature (55). LH is involved in kick-starting reproductive development (56). In boys, LH stimulates the Leydig cells in the testes to promote testosterone production (7) and FSH stimulates testicular growth by promoting Sertoli cell and germ cell proliferation, which may be important for future fertility (57). In girls, FSH initiates ovarian follicle development and estrogen production (46). After mini-puberty, the upper limit of the RI for the hormones from the HPG axis is very low but rises at the onset of puberty, in line with expected physiological maturation (3, 47, 50). In boys, at Tanner stage 5, testosterone RIs seem to reach a plateau at adult RIs, corresponding to the completion of pubertal development. Estradiol RIs in boys show a similar, though less pronounced, pattern. This is consistent with estradiol being primarily a conversion product of testosterone in males (22). In girls, the upper limit of the estradiol RI in girls starts to rise around the age of 8 years, coinciding with the onset of puberty and increasing Tanner stage (9). SHBG, responsible for transporting sex steroids throughout the body (20), shows a different pattern regarding the RIs.

Although we can see clear patterns in RIs of the hormones of the HPG axis over the years and Tanner stages, considerable variation in absolute values has been observed among studies. There are several explanations for these differences. First, there are differences between the analytical methods used to quantify the hormones. The LC–MS/MS methods for the steroid hormones may show some standardization differences: in particular, the immunoassays for LH, FSH, and SHBG are known to show standardization differences. It was not possible to correct for these standardization differences as the methods and their standardization may have changed over time. Because of these differences between immunoassays, it is crucial that laboratories carefully evaluate the specific assay used to quantify hormone concentrations when implementing RIs from the literature. Second, the reliability of the calculated RIs is dependent on the statistical method chosen. In the included studies, a variety of approaches were used to present RIs. Some studies reported median and range (25), which can result in a wide RI. Other studies reported mean ± SD (10), the 5th and 95th percentile (27), or median and 10th and 90th percentile (36), which results in smaller RIs. The statistical methods used to calculate 95% RIs varied, making direct comparison of RIs across studies challenging. It varied from parametric to nonparametric or robust methods or even R packages for continuous RI estimation. A third explanation is the heterogeneity in age categories used to calculate age-specific RIs, for instance, age categories of 1–3 years as opposed to 1–13 years, and the way the Tanner stages were determined. Some studies relied solely on pubic hair growth (PH), while others used testicular volume (TV), genital stage (G), or breast stage (B), to define the Tanner stage. In any case, the examination of Tanner stage is a clinical evaluation and therefore inherently subjective. Moreover, several studies do not report the qualifications of the examiner or the age range of the children at the time of Tanner assessment, ethnic background, or body mass index (BMI). This subjectivity may introduce heterogeneity within groups and consequently affect the RIs. Next, most studies included in this review showed poor sample size per age or Tanner category, as shown in the figures, which certainly leads to greater variability and less reliable RIs. In some cases, Tanner stage categories were combined to obtain larger sample sizes; the same data were then separately presented for each Tanner stage. Finally, in case of the RIs in older girls, there is a lack of information regarding the timing of blood collection in relation to the menstrual cycle. Hormones such as estradiol, LH, and FSH synchronize a woman’s menstrual cycle (58) and the absence of this information results in broader RIs, as samples that are collected throughout the whole menstrual cycle are pooled. RIs defined for different phases of the menstrual cycle would be more precise and narrow.

Despite these limitations, this review provides a valuable overview of the studies published on RIs for hormones of the HPG axis. It highlights the critical need for standardization of methods for hormone quantification, statistical methods to establish RIs, larger and more representative sample sizes, and consistent reporting of age categories, Tanner stage, and menstrual cycle phase. The establishment of RIs is a process which, if improved, could support optimal diagnostic and therapeutic decision-making. We acknowledge, however, the practical and ethical difficulties of collecting 120 samples or more for each partitioned category. In the future, using modern indirect RI methods, such as Refine R (59) or TMC (60), that derive RIs from existing patient data rather than establishing them directly in healthy volunteers, may be helpful to improve the establishment of these RIs. Until there are robust RIs available, the data shown in this review may be used, with caution, to interpret laboratory results of children aged 0–18 years.

## Supplementary materials



## Declaration of interest

The authors have nothing to declare. Anita Boelen is a Senior Editor of *Journal of Molecular Endocrinology*. Anita Boelen was not involved in the review or editorial process for this paper on which she is listed as an author.

## Funding

This work did not receive any specific grant from any funding agency in the public, commercial, or not-for-profit sector.

## Author contribution statement

AO conceived the study, collected and curated the data, performed formal analysis, and wrote the original draft of the manuscript. AB and ACH conceived and supervised the study and wrote, reviewed, and edited the manuscript. JJH wrote, reviewed, and edited the manuscript. SEH conceived the study and wrote, reviewed, and edited the manuscript.

## Generative AI statement

Generative artificial intelligence (AI) tools were used to assist with language editing during the preparation of this manuscript. Specifically, AI Proeftuin (based on OpenAI’s GPT 5.1 model, accessed via an institutional interface) and DeepL Write (DeepL SE, Cologne, Germany; web-based writing assistant, https://www.deepl.com/write) were used to suggest grammatical and stylistic improvements to selected sentences. All AI-edited text was reviewed and finalized by the authors, who take full responsibility for the content of the manuscript.
