# Supplementary material for: A literature overview of age- and Tanner stage-specific reference intervals for sex hormones for children aged 0–18 years
Source: J Mol Endocrinol. 2026 Aug 21;77(2):e260037. doi: 10.1530/JME-26-0037 (PMC13506540; doi:10.1530/JME-26-0037)
Supplement: Supplementary file 1 [file JME-26-0037_supplementary_tables.pdf]

Supplemental Table 1. Sample size per age-category for testosterone reference intervals. Sample size indication - red: ≤38, orange: 39 < 120, green: ≥120, grey: no information available, white: no reference interval determined.

Article	*Boys (n)*	*Sample size quality*	*Girls (n)*	*Sample size quality*
Kulle et al. 2010 (< 1 week)	8		6	
Kyriakopoulou et al. 2013 (0 - 14 days)	18		13	
Busch et al. 2025 (0 - 30 days)	?		?	
Kulle et al. 2010 (2 weeks - 2 months)	19		23	
Busch et al. 2025 (31 - 180 days)	?		?	
Johannsen et al. 2018 (2.0 - 3.5 months)	251		165	
Kulle et al. 2010 (3 - 5 months)	12		10	
Johannsen et al. 2018 (3.5 - 5.0 months)	175		125	
Rauh et al. 2006 (<0.5 years)	25		15	
Busch et al. 2025 (181 - 400 days)	?		?	
Kyriakopoulou et al. 2013 (15 days - <1 year)	34		28	
Bae et al. 2019 (<1 years)	124		156	
Kulle et al. 2010 (6 - 11 months)	4		10	
Kushnir et al. 2010 (6 - 24 months)	123		92	
Kulle et al. 2010 (1 - 3 years)	9		9	
Rauh et al. 2006 (1 - 6 years)	41		29	
Salameh et al. 2010 (1 - <6 years)	?		?	
Bae et al. 2019 (1 - 10 years)	855		602	
Kyriakopoulou et al. 2013 (1 - <13 years)	138		138	
Kushnir et al. 2010 (2 - 3 years)	125		126	
Soldin et al. 2009 (0 - 5 years)			127	
Soldin et al. 2009 (0 - 6 years)	158			
Kushnir et al. 2010 (4 - 5 years)	125		127	
Kulle et al. 2010 (4 - 6 years)	10		13	
Frederiksen et al. 2024 (5 years)	?		?	
Kushnir et al. 2010 (6 - 7 years)	125		131	
Salameh et al. 2010 (6 - <8 years)	?		?	
Soldin et al. 2009 (6 - 9 years)			171	
Madsen et al. 2019 (6 to <10 years)	169			
Frederiksen et al. 2024 (7.5 years)	?		?	
Soldin et al. 2009 (7 - 9 years)	148			
Kulle et al. 2010 (7 - 9 years)	10		14	
Kushnir et al. 2010 (7 - 9 years)	206		206	
Rauh et al. 2006 (7 - 12 years)	29		33	
Salameh et al. 2010 (8 - <11 years)	?		?	
Frederiksen et al. 2024 (10 years)	?		?	
Kushnir et al. 2010 (10 - 11 years)	140		148	
Soldin et al. 2009 (10 - 12 years)	188			
Kulle et al. 2010 (10 - 12 years)	18		17	
Madsen et al. 2019 (10 to <13 years)	137			

Soldin et al. 2009 (10 - 14 years)			190	
Bae et al. 2019 (10 - 15 years)	730		622	
Salameh et al. 2010 (11 - <12 years)	?		?	
Frederiksen et al. 2024 (12.5 years)	?		?	
Kushnir et al. 2010 (12 - 13 years)	143		142	
Salameh et al. 2010 (12 - <14 years)	?		?	
Soldin et al. 2009 (13 - 14 years)	245			
Rauh et al. 2006 (13 - 15 years)	18		10	
Kulle et al. 2010 (13 - 15 years)	26		19	
Kyriakopoulou et al. 2013 (13 - <16 years)	18		17	
Madsen et al. 2019 (13 to <16 years)	108			
Kushnir et al. 2010 (14 - 15 years)	141			
Salameh et al. 2010 (14 - <18 years)	?		?	
Frederiksen et al. 2024 (15 years)	?		?	
Soldin et al. 2009 (15 - 16 years)	209		175	
Bae et al. 2019 (15 - 20 years)	336		319	
Kushnir et al. 2010 (16 - 17 years)	136		138	
Rauh et al. 2006 (16 - 18 years)	12		11	
Kulle et al. 2010 (16 - 18 years)	21		10	
Kyriakopoulou et al. 2013 (16 - <19 years)	30		31	
Soldin et al. 2009 (17 - 18 years)			123	
Soldin et al. 2009 (17 - 19 years)	123			
Frederiksen et al. 2024 (20 years)	?		?	

Supplemental Table 2. Sample size per age-category for estradiol reference intervals. Sample size indication - red: ≤38, orange: 39 < 120, green: ≥120, grey: no information available.

Article	***Boys (n)***	***Sample size quality***	***Girls (n)***	***Sample size quality***
Bae et al. 2019 (<1 years)	124		156	
Won et al. 2023 (<1 years)#	39		41	
Di Meo et al. 2023 (<1 years)	21		22	
*Kulle et al. 2024 (0 - 1 years)**	52		72	
*Kulle et al. 2024 (1 - 2 years)**	12		17	
*Kulle et al. 2024 (2 - 3 years)**	7		20	
*Kulle et al. 2024 (3 - 4 years)**	6		21	
*Kulle et al. 2024 (4 - 5 years)**	13		25	
Bae et al. 2019 (1 - 5 years)	206		47	
Won et al. 2023 (1 - 5 years)#	44		44	
Di Meo et al. 2023 (1 - 10 years)	40		44	
Frederiksen et al. 2020 (5 years)	?		?	
*Kulle et al. 2024 (5 - 6 years)**	11		45	
Bae et al. 2019 (5 - 10 years)	649		557	
*Kulle et al. 2024 (6 - 7 years)**	16		41	
Won et al. 2023 (6 - 9 years)#	48		88	

Frederiksen et al. 2020 (7.5 years)	?		?	
*Kulle et al. 2024 (7 - 8 years)**	16		89	
*Kulle et al. 2024 (8 - 9 years)**	25		153	
*Kulle et al. 2024 (9 - 10 years)**	37		138	
Frederiksen et al. 2020 (10 years)	?		?	
Won et al. 2023 (10 - 11 years)#	45		41	
Kulle et al. 2024 (10 - 11 years)	35		106	
Di Meo et al. 2023 (10 - 13 years)	40		42	
Bae et al. 2019 (10 - 15 years)	731		629	
*Kulle et al. 2024 (11 - 12 years)**	34		99	
Frederiksen et al. 2020 (12.5 years)	?		?	
*Kulle et al. 2024 (12 - 13 years)**	48		67	
Won et al. 2023 (12 - 14 years)#	41		62	
*Kulle et al. 2024 (13 - 14 years)**	74		79	
Di Meo et al. 2023 (13 - 15 years)	45		40	
*Kulle et al. 2024 (14 - 15 years)**	54		61	
Frederiksen et al. 2020 (15 years)	?		?	
*Kulle et al. 2024 (15 - 16 years)**	44		56	
Won et al. 2023 (15 - 17 years)#	59		40	
Di Meo et al. 2023 (15 - 19 years)	42		53	
Bae et al. 2019 (15 - 20 years)	335		321	
*Kulle et al. 2024 (16 - 17 years)**	30		60	
*Kulle et al. 2024 (17 - 18 years)**	29		32	
Frederiksen et al. 2020 (20 years)	?		?	

Supplemental Table 3. Sample size per age-category for Luteinizing Hormone (LH) reference intervals. Sample size indication - red: ≤38, orange: 39 < 120, green: ≥120, white: no reference interval determined.

Article	***Boys (n)***	***Sample size quality***	***Girls (n)***	***Sample size quality***
Higgins et al. 2018 (0 to <6 months)	62		62	
Bohn et al. 2019 (0 to <6 months)	40		49	
Karbasy et al. 2016 (0 to <1 year)	110		63	
Bohn et al. 2021 (0 to <1 years)	27		25	
Soldin et al. 2005 (0 > 6 years)			48	
Soldin et al. 2005 (0 > 13 years)	47			
Johannsen et al. 2018 (2.0 - 3.5 months)	581		432	
Konforte et al. 2013 (4 days–<3 months)	51		51	
Elmlinger et al. 2002 (16 days - 3 years)	42		44	
Konforte et al. 2013 (3 months–<1 year)	125		99	
Johannsen et al. 2018 (3.5 - 5.0 months)	166		110	
Higgins et al. 2018 (6 months to <11 years)	319		319	
Bohn et al. 2019 (6 months to <11 years)	245		245	

Supplemental Table 4. Sample size per age-category for FSH reference intervals. Sample size indication - red: ≤38, orange: 39 < 120, green: ≥120, white: no reference interval determined.

Article	*Boys (n)*	*Sample size quality*	*Girls (n)*	*Sample size quality*
Koskas et al. 2007 (1-3 years)	17	[red]	19	[red]
Karbasy et al. 2016 (1 to <5 years)	114	[orange]	114	[orange]
Zec et al. 2012 (≥ 1 to < 8 years)	302	[green]	305	[green]
Konforte et al. 2013 (1–<10 years)	336	[green]	336	[green]
Bohn et al. 2021 (1 to <11 years)	175	[green]	175	[green]
Elmlinger et al. 2002 (4-6 years)	28	[red]	23	[red]
Koskas et al. 2007 (4-8 years)	24	[red]	22	[red]
Karbasy et al. 2016 (5 to <10 years)	131	[green]	131	[green]
Madsen et al. 2019 (6 to < 10 years)	168	[green]		
Soldin et al. 2005 (6 > 11 years)			106	[orange]
Elmlinger et al. 2002 (7-8 years)	26	[red]	24	[red]
Zec et al. 2012 (≥ 8 to < 11 years)	118	[orange]	116	[orange]
Elmlinger et al. 2002 (9-10 years)	31	[red]	40	[orange]
Madsen et al. 2019 (10 to < 13 years)	135	[green]		[green]
Konforte et al. 2013 (10–<13 years)	210	[green]	210	[orange]
Karbasy et al. 2016 (10 to <14 years)	56	[orange]	51	[red]
Elmlinger et al. 2002 (11 years)	22	[red]	23	[orange]
Higgins et al. 2018 (11 to <13 years)	82	[orange]	84	[orange]
Bohn et al. 2019 (11 to <14 years)	53	[orange]	62	[red]
Bohn et al. 2021 (11 to <14 years)	43	[orange]	33	[orange]
Soldin et al. 205 (11 < 15 years)			87	[orange]
Elmlinger et al. 2002 (12 years)	17	[red]	18	[red]
Elmlinger et al. 2002 (13 years)	21	[red]	25	[red]
Konforte et al. 2013 (13–<15 years)	73	[orange]	60	[orange]
Higgins et al. 2018 (13 to <16 years)	91	[orange]	81	[orange]
Madsen et al. 2019 (13 to <16 years)	107	[orange]		
Soldin et al. 2005 (13 > 19 years)	62	[orange]		
Elmlinger et al. 2002 (14 years)	32	[red]	30	[red]
Karbasy et al. 2016 (14 to <19 years)	70	[orange]	67	[orange]
Bohn et al. 2021 (14 to <19 years)	66	[orange]	69	[orange]
Bohn et al. 2019 (14 to <19 years)	86	[orange]	83	[orange]
Elmlinger et al. 2002 (15 years)	40	[orange]	48	[orange]
Konforte et al. 2013 (15–<17 years)	66	[orange]	63	[orange]
Soldin et al. 2005 (15 > 19 years)			111	[orange]
Elmlinger et al. 2002 (16 years)	31	[red]	40	[orange]
Higgins et al. 2018 (16 to <19 years)	87	[orange]	85	[orange]
Elmlinger et al. 2002 (17 years)	22	[red]	30	[red]
Konforte et al. 2013 (17–<19 years)	69	[orange]	51	[orange]
Elmlinger et al. 2002 (18-19 years)	8	[red]	12	[red]

Elmlinger et al. 2002 (1-7 days)	28	17
Elmlinger et al. 2002 (8 - 15 days)	20	20
Higgins et al. 2018 (0 to <1 year)	44	64
Konforte et al. 2013 (0 days - <1 year)	142	
Karbasy et al. 2016 (0 to <1 year)	105	53
Bohn et al. 2019 (0 to <1 year)	44	58
Bohn et al. 2021 (0 to <1 years)	27	31
Soldin et al. 2005 (0 > 6 years)		51
Johannsen et al. 2018 (2.0 - 3.5 months)	578	435
Elmlinger et al. 2002 (16 days - 3 years)	42	44
Konforte et al. 2013 (30 days - <1 year)		118
Johannsen et al. 2018 (3.5 - 5.0 months)	165	111
Koskas et al. 2007 (1-3 years)	18	19
Zec et al. 2012 (≥ 1 to < 4 years)		124
Konforte et al. 2013 (1 - <5 years)	67	
Zec et al. 2012 (≥ 1 to < 8 years)	303	
Karbasy et al. 2016 (1 to < 9 years)	130	114
Higgins et al. 2018 (1 to < 9 years)	127	97
Bohn et al. 2019 (1 to <9 year)	80	67
Bohn et al. 2021 (1 to <9 year)	86	79
Konforte et al. 2013 (1 - <9 years)		119
Elmlinger et al. 2002 (4-6 years)	28	23
Koskas et al. 2007 (4-8 years)	26	22
Zec et al. 2012 (≥ 4 to < 8 years)		156
Konforte et al. 2013 (5 - <10 years)	118	
Madsen et al. 2019 (6 to < 10 years)	169	
Soldin et al. 2005 (6 > 11 years)		106
Elmlinger et al. 2002 (7-8 years)	26	24
Zec et al. 2012 (≥ 8 to < 11 years)	117	107
Elmlinger et al. 2002 (9-10 years)	31	40
Konforte et al. 2013 (9 - <11 years)		74
Karbasy et al. 2016 (9 to <12 years)	41	41
Higgins et al. 2018 (9 to < 12 years)	87	85
Bohn et al. 2019 (9 to <12 years)	42	55
Bohn et al. 2021 (9 to <12 years)	43	45
Madsen et al. 2019 (10 to < 13 years)	137	
Konforte et al. 2013 (10 - <13 years)	110	
Elmlinger et al. 2002 (11 years)	22	23
Soldin et al. 2005 (11 > 15 years)		86
Konforte et al. 2013 (11 - <19 years)		275
Elmlinger et al. 2002 (12 years)	17	18
Bohn et al. 2019 (12 to <19 years)	125	122
Bohn et al. 2021 (12 to <19 years)	94	103
Karbasy et al. 2016 (12 to <19 years)	94	100
Higgins et al. 2018 (12 to < 19 years)	125	142
Elmlinger et al. 2002 (13 years)	21	25

Article	Boys (n)	Sample size quality	Girls (n)	Sample size quality
Madsen et al. 2019 (13 to <16 years)	108	orange		
Soldin et al. 2005 (13 > 19 years)	62	orange		
Konforte et al. 2013 (13 - <19 years)	202	green		
Elmlinger et al. 2002 (14 years)	32	red	30	red
Elmlinger et al. 2002 (15 years)	40	orange	48	orange
Soldin et al. 2005 (15 > 19 years)			112	orange
Elmlinger et al. 2002 (16 years)	31	red	40	orange
Elmlinger et al. 2002 (17 years)	22	red	30	red
Elmlinger et al. 2002 (18-19 years)	8	red	12	red

Supplemental Table 5. Sample size per age-category for SHBG reference intervals. Sample size indication - red: ≤38, orange: 39 < 120, green: ≥120, white: no reference interval determined.

Article	***Boys (n)***	***Sample size quality***	***Girls (n)***	***Sample size quality***
Elmlinger et al. 2002 (1-7 days)	28	red	17	red
Elmlinger et al. 2002 (8 - 15 days)	20	red	20	red
Karbasy et al. 2016 (<1 month)	59	orange	59	orange
Bohn et al. 2019 (<1 month)	50	orange	50	orange
Konforte et al. 2013 (4 days - 1 month)	113	orange	113	orange
Elmlinger et al. 2002 (16 days - 3 years)	42	orange	44	orange
Konforte et al. 2013 (1 month -1 year)	239	green	239	green
Karbasy et al. 2016 (1 month to <1 year)	128	green	128	green
Bohn et al. 2019 (1 month to <13 years)	378	green	378	green
Johannsen et al. 2018 (2.0 - 3.5 months)	579	green	427	green
Johannsen et al. 2018 (3.5 - 5.0 months)	162	green	110	orange
Konforte et al. 2013 (1 - <8 years)	212	green	236	green
Karbasy et al. 2016 (1 to < 8 years)	236	green	212	green
Zec et al. 2012 (≥ 1 to < 11 years)	179	green	109	orange
Elmlinger et al. 2002 (4-6 years)	28	red	23	red
Madsen et al. 2019 (6 to < 10 years)	169	green		
Elmlinger et al. 2002 (7-8 years)	26	red	24	red
Kushnir et al. 2006 (7 - 9 years)	105	orange	103	orange
Elmlinger et al. 2002 (9-10 years)	31	red		
Konforte et al. 2013 (8 - <11 years)	184	green	184	green
Karbasy et al. 2016 (8 to <11 years)	80	orange	80	orange
Elmlinger et al. 2002 (9-10 years)			40	orange
Kushnir et al. 2006 (10 - 11 years)	68	orange	69	orange
Madsen et al. 2019 (10 to < 13 years)	137	green		
Elmlinger et al. 2002 (11 years)	22	red	23	red
Konforte et al. 2013 (11 - <13 years)	57	orange	158	green
Karbasy et al. 2016 (11 to <13 years)	158	green	57	orange
Elmlinger et al. 2002 (12 years)	17	red	18	red
Kushnir et al. 2006 (12 - 13 years)	69	orange	69	orange
Elmlinger et al. 2002 (13 years)	21	red	25	red

Bohn et al. 2019 (13 to <15 year)	59		160	
Konforte et al. 2013 (13 - <15 years)	160		59	
Madsen et al. 2019 (13 to < 16 years)	108			
Karbasy et al. 2016 (13 to <19 years)	84		85	
Elmlinger et al. 2002 (14 years)	32		30	
Kushnir et al. 2006 (14 - 15 years)	70		70	
Elmlinger et al. 2002 (15 years)	40		48	
Konforte et al. 2013 (15 - <17 years)			64	
Konforte et al. 2013 (15 - <19 years)	129			
Bohn et al. 2019 (15 to <19 years)	72		70	
Elmlinger et al. 2002 (16 years)	31		40	
Kushnir et al. 2006 (16 - 17 years)	70		70	
Elmlinger et al. 2002 (17 years)	22		30	
Konforte et al. 2013 (17 - <19 years)			52	
Elmlinger et al. 2002 (18-19 years)	8		12	

Supplemental table 6. Sample size per Tanner stage for testosterone, estradiol, LH, FSH and SHBG reference intervals. Sample size indication - red: ≤38, orange: 39 < 120, green: ≥120, grey: no information available.

Testosterone

Boys: ***Article***	***n***	***Sample size quality***	Girls: ***Article***	***n***	***Sample size quality***
		**Tanner I**			
Ankarberg-Lindgren et al. 2020	20		*Ankarberg-Lindgren et al. 2020*	20	
Bae et al. 2019	266		*Bae et al. 2019*	266	
Madsen et al. 2019	249		*Ankarberg-Lindgren et al. 2018*	16	
Ankarberg-Lindgren et al. 2018	9		*Soeborg et al. 2014*	122	
Soeborg et al. 2014	215		*Kushnir et al. 2010*	296	
Kushnir et al. 2010	278		*Kulle et al. 2010*	14	
Kulle et al. 2010	13		*Salameh et al. 2010*	?	
Salameh et al. 2010	?		*Kushnir et al. 2006*	147	
Kushnir et al. 2006	143				
		**Tanner II**			
Ankarberg-Lindgren et al. 2020	42		*Ankarberg-Lindgren et al. 2020*	42	
Bae et al. 2019	75		*Bae et al. 2019*	75	
Madsen et al. 2019	90		*Ankarberg-Lindgren et al. 2018*	15	
Ankarberg-Lindgren et al. 2018	9		*Soeborg et al. 2014*	32	
Soeborg et al. 2014	109		*Kushnir et al. 2010*	120	
Kushnir et al. 2010	131		*Kulle et al. 2010*	10	
Kulle et al. 2010	11		*Salameh et al. 2010*	?	
Salameh et al. 2010	?		*Kushnir et al. 2006*	66	
Kushnir et al. 2006	66				

**Tanner III**					
Ankarberg-Lindgren et al. 2020	20	[red]	*Ankarberg-Lindgren et al. 2020*	20	[red]
Bae et al. 2019	63	[amber]	*Bae et al. 2019*	63	[amber]
Madsen et al. 2019	90	[amber]	*Ankarberg-Lindgren et al. 2018*	13	[red]
Ankarberg-Lindgren et al. 2018	14	[red]	*Soeborg et al. 2014*	32	[red]
Soeborg et al. 2014	52	[amber]	*Kushnir et al. 2010*	135	[green]
Kushnir et al. 2010	140	[green]	*Kulle et al. 2010*	10	[red]
Kulle et al. 2010	11	[red]	*Salameh et al. 2010*	?	[grey]
Salameh et al. 2010	?	[grey]	*Kushnir et al. 2006*	85	[amber]
Kushnir et al. 2006	65	[amber]			
**Tanner IV**					
Ankarberg-Lindgren et al. 2020	20	[red]	*Ankarberg-Lindgren et al. 2020*	20	[red]
Bae et al. 2019	73	[amber]	*Bae et al. 2019*	73	[amber]
Madsen et al. 2019	90	[amber]	*Ankarberg-Lindgren et al. 2018*	16	[red]
Ankarberg-Lindgren et al. 2018	9	[red]	*Soeborg et al. 2014*	132	[green]
Soeborg et al. 2014	37	[red]	*Kushnir et al. 2010*	205	[green]
Kushnir et al. 2010	204	[green]	*Kulle et al. 2010*	11	[red]
Kulle et al. 2010	10	[red]	*Salameh et al. 2010*	?	[grey]
Salameh et al. 2010	?	[grey]	*Kushnir et al. 2006*	77	[amber]
Kushnir et al. 2006	98	[amber]			
**Tanner V**					
Ankarberg-Lindgren et al. 2020	20	[red]	*Ankarberg-Lindgren et al. 2020*	20	[red]
Ankarberg-Lindgren et al. 2020	20	[amber]	*Bae et al. 2019*	114	[amber]
Bae et al. 2019	114	[amber]	*Ankarberg-Lindgren et al. 2018*	16	[red]
Ankarberg-Lindgren et al. 2018	16	[red]	*Soeborg et al. 2014*	79	[amber]
Soeborg et al. 2014	79	[green]	*Kushnir et al. 2010*	205	[green]
Kushnir et al. 2010	205	[green]	*Kulle et al. 2010*	12	[red]
Kulle et al. 2010	12	[red]	*Salameh et al. 2010*	?	[grey]
Salameh et al. 2010	?	[grey]	*Kushnir et al. 2006*	123	[green]
Kushnir et al. 2006	123	[amber]			

Estradiol

<u>*Boys*</u> / <u>*Girls*</u>

Article	***n***	***Sample size quality***	***Article***	***n***	***Sample size quality***
**Tanner I**					
Bae et al. 2019	266	[green]	*Kulle et al. 2024*	147	[green]
Ankarberg-Lindgren et al. 2018	20	[red]	*Madsen et al. 2020*	260	[green]
			Bae et al. 2019	224	[green]
			Ankarberg-Lindgren et al. 2018	23	[red]
**Tanner II**					
Bae et al. 2019	75	[amber]	*Kulle et al. 2024*	192	[green]
Ankarberg-Lindgren et al. 2018	42	[amber]	*Madsen et al. 2020*	69	[amber]
			Bae et al. 2019	99	[amber]
			Ankarberg-Lindgren et al. 2018	24	[red]
**Tanner III**					
Bae et al. 2019	63	[amber]	*Kulle et al. 2024*	178	[green]

Ankarberg-Lindgren et al. 2018	20		*Madsen et al. 2020*	63	
			Bae et al. 2019	74	
			Ankarberg-Lindgren et al. 2018	17	
**Tanner IV**					
Bae et al. 2019	73		*Kulle et al. 2024*	98	
Ankarberg-Lindgren et al. 2018	20		*Madsen et al. 2020*	73	
			Bae et al. 2019	68	
			Ankarberg-Lindgren et al. 2018	21	
**Tanner V**					
Bae et al. 2019	114		*Kulle et al. 2024*	168	
Ankarberg-Lindgren et al. 2018	20		*Madsen et al. 2020*	76	
			Bae et al. 2019	90	
			Ankarberg-Lindgren et al. 2018	21	

LH

Boys | Girls

**Article**	***n***	***Sample size quality***	**Article**	***n***	***Sample size quality***
**Tanner I**					
Madsen et al. 2019	246		*Madsen et al. 2020*	269	
Konforte et al. 2013	25		*Konforte et al. 2013*	23	
Koskas et al. 2007	32		*Koskas et al. 2007*	17	
Elmlinger et al. 2002	33		*Elmlinger et al. 2002*	28	
			Sehested et al. 2000	124	
**Tanner II**					
Madsen et al. 2019	90		*Madsen et al. 2020*	73	
Konforte et al. 2013	36		*Konforte et al. 2013*	24	
Koskas et al. 2007	18		*Koskas et al. 2007*	15	
Elmlinger et al. 2002	25		*Elmlinger et al. 2002*	25	
			Sehested et al. 2000	40	
**Tanner III**					
Madsen et al. 2019	90		*Madsen et al. 2020*	71	
Konforte et al. 2013	21		*Konforte et al. 2013*	29	
Koskas et al. 2007	15		*Koskas et al. 2007*	19	
Elmlinger et al. 2002	50		*Elmlinger et al. 2002*	47	
			Sehested et al. 2000	29	
**Tanner IV**					
Madsen et al. 2019	90		*Madsen et al. 2020*	90	
Konforte et al. 2013	52		*Konforte et al. 2013*	60	
Koskas et al. 2007	14		*Koskas et al. 2007*	12	
Elmlinger et al. 2002	39		*Elmlinger et al. 2002*	31	
			Sehested et al. 2000	39	
**Tanner V**					
Madsen et al. 2019	63		*Madsen et al. 2020*	89	
Konforte et al. 2013	17		*Konforte et al. 2013*	34	
Koskas et al. 2007	28		*Koskas et al. 2007*	27	
Elmlinger et al. 2002	38		*Elmlinger et al. 2002*	65	

Article	n	Sample size quality	Article	n	Sample size quality
			Sehested et al. 2000	170	green

FSH

<u>*Boys*</u> / <u>*Girls*</u>

Article	***n***	***Sample size quality***	***Article***	***n***	***Sample size quality***
**Tanner I**					
Madsen et al. 2019	249	green	*Madsen et al. 2020*	276	green
Konforte et al. 2013	21	red	*Konforte et al. 2013*	22	red
Koskas et al. 2007	19	red	*Koskas et al. 2007*	34	red
Elmlinger et al. 2002	33	red	*Elmlinger et al. 2002*	28	red
			Sehested et al. 2000	124	green
**Tanner II**					
Madsen et al. 2019	90	orange	*Madsen et al. 2020*	73	orange
Konforte et al. 2013	34	red	*Konforte et al. 2013*	26	red
Koskas et al. 2007	16	red	*Koskas et al. 2007*	19	red
Elmlinger et al. 2002	25	red	*Elmlinger et al. 2002*	25	red
			Sehested et al. 2000	40	orange
**Tanner III**					
Madsen et al. 2019	90	orange	*Madsen et al. 2020*	71	orange
Konforte et al. 2013	22	red	*Konforte et al. 2013*	30	red
Koskas et al. 2007	19	red	*Koskas et al. 2007*	15	red
Elmlinger et al. 2002	50	orange	*Elmlinger et al. 2002*	47	orange
			Sehested et al. 2000	29	red
**Tanner IV**					
Madsen et al. 2019	90	orange	*Madsen et al. 2020*	90	orange
Konforte et al. 2013	51	orange	*Konforte et al. 2013*	61	orange
Koskas et al. 2007	14	red	*Koskas et al. 2007*	14	red
Elmlinger et al. 2002	39	orange	*Elmlinger et al. 2002*	31	red
			Sehested et al. 2000	39	orange
**Tanner V**					
Madsen et al. 2019	64	orange	*Madsen et al. 2020*	90	orange
Konforte et al. 2013	71	orange	*Konforte et al. 2013*	36	red
Koskas et al. 2007	30	red	*Koskas et al. 2007*	30	red
Elmlinger et al. 2002	38	red	*Elmlinger et al. 2002*	65	orange
			Sehested et al. 2000	170	green

SHBG

<u>*Boys*</u> / <u>*Girls*</u>

Article	***n***	***Sample size quality***	***Article***	***n***	***Sample size quality***
**Tanner I**					
Madsen et al. 2019	22	red	*Madsen et al. 2020*	269	green
Konforte et al. 2013	143	green	*Konforte et al. 2013*	147	green
Kushnir et al. 2006	249	green	*Kushnir et al. 2006*	28	red
Elmlinger et al. 2002	33	red	*Elmlinger et al. 2002*	22	red
**Tanner II**					
Madsen et al. 2019	26	red	*Madsen et al. 2020*	73	orange

Konforte et al. 2013	66		*Konforte et al. 2013*	66	
Kushnir et al. 2006	90		*Kushnir et al. 2006*	25	
Elmlinger et al. 2002	25		*Elmlinger et al. 2002*	26	
**Tanner III**					
Madsen et al. 2019	31		*Madsen et al. 2020*	71	
Konforte et al. 2013	65		*Konforte et al. 2013*	85	
Kushnir et al. 2006	90		*Kushnir et al. 2006*	47	
Elmlinger et al. 2002	50		*Elmlinger et al. 2002*	31	
**Tanner IV**					
Madsen et al. 2019	62		*Madsen et al. 2020*	90	
Konforte et al. 2013	98		*Konforte et al. 2013*	77	
Kushnir et al. 2006	90		*Kushnir et al. 2006*	31	
Elmlinger et al. 2002	39		*Elmlinger et al. 2002*	62	
**Tanner V**					
Madsen et al. 2019	33		*Madsen et al. 2020*	90	
Konforte et al. 2013	42		*Konforte et al. 2013*	123	
Kushnir et al. 2006	64		*Kushnir et al. 2006*	65	
Elmlinger et al. 2002	38		*Elmlinger et al. 2002*	33	
